# Supplementary material for: Osteopathic Treatment and Evaluation in the Clinical Setting of Childhood Hematological Malignancies
Source: Cancers (Basel). 2021 Dec 16;13(24):6321. doi: 10.3390/cancers13246321 (PMC8699143; doi:10.3390/cancers13246321)
Supplement: Supplementary file 1 [file cancers-13-06321-s001.zip › cancers-1498992-supplementary.pdf]

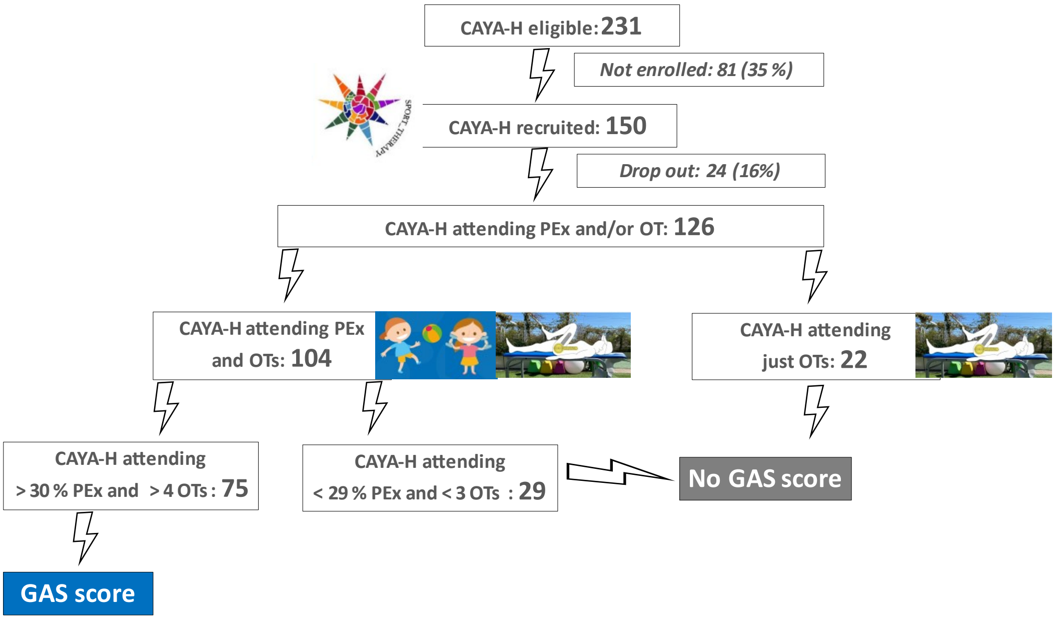

Figure S1: Participants' flow chart, from the eligibility number of participants to those with a GAS score (see the methods section for further explanation). CAYA-H: Children, adolescents and young adults with hematological malignancies; PEx: precision-based exercise program; OT: osteopathic treatment. GAS: goal attainment scaling.
